# Feasibility of Self-Reported Surveillance After Catheter Ablation for Atrial Fibrillation Using A Mobile Application

**DOI:** 10.1016/j.jacadv.2026.102625

**Published:** 2026-03-25

**Authors:** Adi Elias, Jessica N. Holtzman, Manuella L. Djomaleu, James V. Freeman, Andrea Natale, Jonathan C. Hsu, Wendy S. Tzou, Lohit Garg, Jonathan W. Dukes, Daniel H. Cooper, William Gionfriddo, Isaac R. Whitman, Nino Isakadze, David D. Spragg, Danish Iltaf Satti, David G. Rosenthal, Eric M. Riles, Mellanie True Hills, Debbe McCall, Gabrielle C. Montenegro, Hannah H. Oo, Dylan A. Lowe, Edward P. Gerstenfeld, Joshua D. Moss, Thomas A. Dewland, Henry H. Hsia, Randall J. Lee, Zian H. Tseng, Vasanth Vedantham, Gregory M. Marcus

**Affiliations:** a Division of Cardiology, Department of Medicine, University of California-San Francisco, San Francisco, USA; b Section of Cardiovascular Medicine, Yale University, New Haven, USA; c Texas Cardiac Arrhythmia Institute, St David's Medical Center, Austin, USA; d Division of Cardiology, Department of Biomedicine and Prevention, University of Tor Vergata, Rome, Italy; e Department of Cardiology, University of California-San Diego, San Diego, USA; f Division of Cardiology, University of Colorado Anschutz Medical Center, Aurora, USA; g Division of Cardiology, Community Memorial Hospital, Ventura, USA; h Cardiovascular Division, Washington University, St. Louis, USA; i Division of Cardiology, Saint Francis Hospital, Hartford, USA; j Section of Cardiology, Department of Medicine, Lewis Katz School of Medicine at Temple University, Philadelphia, USA; k Division of Cardiology, Johns Hopkins Hospital, Baltimore, USA; l Department of Internal Medicine, University of Washington Medical Center, Seattle, USA; m Division of Cardiology, UCHealth Heart and Vascular Clinic, Loveland, USA; n StopAfib.org, Decatur, Texas, USA; o Atrial fibrillation patient

**Keywords:** atrial fibrillation, catheter ablation, mobile-application, quality assurance, self-reports

## Abstract

**Background:**

The effectiveness of catheter ablation for atrial fibrillation (AF) can only be fully assessed months after the procedure, and many complications do not present until after hospital discharge. Readily available means to monitor patients for quality assurance are hampered by the impracticality of following patients after they leave referral centers.

**Objectives:**

The objective of the study was to determine if a mobile app-based platform that interfaced directly with AF ablation patients using their smartphones might be feasible.

**Methods:**

In the VIBRANT-AF (Volunteers to Investigate Best Results for Ablation and Novel Therapies for Atrial Fibrillation) study, AF catheter ablation patients were monitored with baseline and weekly SMS messages accompanied by mobile application–based questionnaires using the Eureka digital research platform.

**Results:**

Among 493 participants enrolled from 17 sites, 4% reported an emergency department visit related to AF or the catheter ablation procedure. Among 236 followed for 1 year, participants completed a median of 25 (IQR: 7-42) electronic visits. A 2-fold increase in response rate was observed among those who completed at least 1 of the first 2 weeks of surveys. Recurrent AF at 1 year was reported in 30% of participants. The validated Atrial Fibrillation Effect on Quality of Life score improved from baseline to 6 months, with sustained improvement at 12 months. Validation of self-reported AF and complications in a subset of the cohort demonstrated substantial agreement with KardiaMobile electrocardiograms and electronic health record data, respectively.

**Conclusions:**

Direct patient-facing, mobile application–based surveillance is feasible following catheter AF ablation, with self-reported rates of effectiveness and complications similar to previous reports using conventional means. (Volunteers to Investigate Best Results for Ablation and Novel Therapies for Atrial Fibrillation [VIBRANT-AF]; NCT05504356)

The prevalence of atrial fibrillation (AF) is rising worldwide,^1^ and catheter ablation for AF is the most commonly performed cardiac electrophysiology procedure.^2^, ^3^, ^4^ It is estimated that by 2030, up to 30 million individuals worldwide will have undergone catheter ablation for AF.^5^

AF ablation may be the most effective treatment for AF, but it is also the highest-risk approach to managing the disease.^6^, ^7^, ^8^, ^9^ Although randomized trials demonstrate efficacy in AF eradication superior to any other method,^10^, ^11^, ^12^ the data describing the effectiveness and safety of the procedure arise almost exclusively from randomized trials or observational studies from high-volume centers staffed by experts.^3^^,^^8^^,^^13^

An effective and affordable tool is required to monitor patient outcomes and complications following their leave of referral centers performing this common catheter ablation procedure.^14^^,^^15^ However, readily available means to monitor patients for quality assurance/improvement (QA/QI) are hampered by the impracticality of following patients, particularly given the high costs of research personnel and the inconvenience of returning to referral centers.

The great majority of Americans (even the elderly) now own a smartphone,^16^ opening up the possibility of regular engagement via text messages, mobile applications, and connected devices. The VIBRANT-AF (Volunteers to Investigate Best Results for Ablation and Novel Therapies for Atrial Fibrillation) study was conducted to leverage smartphones as a means to longitudinally follow patients after AF ablation procedures.

In the current analysis, we sought to determine if a mobile app-based platform that interfaced directly with AF ablation patients might be feasible to assess procedural outcomes and adverse events in a “real-world” setting.

## Methods

VIBRANT-AF is an investigator-initiated, observational longitudinal registry funded by the National Institutes of Health and supported by the National Cardiovascular Disease Registry and the American College of Cardiology. The mobile application (app) was constructed using the Eureka Digital Research Platform (University of California-San Francisco [UCSF]).^17^ The study was approved by the UCSF Institutional Review Board, and all participants provided electronic consent via the Eureka mobile app.

## Population

Patients aged ≥18 undergoing catheter ablation for AF who owned a smart phone (iOS and/or Android) were eligible for the study. Patients at participating sites were handed postcards with quick response codes and text-backs unique to each site that provided deep links to the VIBRANT AF app (Videos 1 and 2). Study information was provided and informed consent obtained entirely via the app; some participants through word-of-mouth or internet, patient advocacy groups (author M.T.H.), and via social-media (author D.M.). Participants unable to consent for themselves or those unable to read, speak, or comprehend English or Spanish were excluded from the study.

Participants completed baseline questionnaires and then received weekly invitations accompanied by links to electronic surveys beginning within 1 week of their scheduled AF ablation procedure. Alerts were initially sent via a mobile app push notification, which was escalated to a text message within an hour if the survey was not completed. If the surveys, comprising that week’s “evisit,” were not completed within 3 days, another reminder was sent (again accompanied by an electronic link to the survey).

### The mobile app

The app was built using the Eureka Digital Research platform, a mobile-health–based research platform supported by a U-award from the National Institutes of Health to facilitate technology-based research. All Eureka personnel are based at UCSF. The mobile app was custom built for the study and operates on both iOS and Android devices. The app includes a programmable messaging queue sent by in-app notifications and/or short message service text message. The app delivers surveys at a programmable cadence, with triggered responses depending on prior responses.

### Surveys

Baseline surveys collected information regarding patient demographics, education and income, AF characterization, medical conditions, habits (smoking, caffeine, alcohol, and physical activity), and whether the participant owned a commercially available electrocardiogram (ECG) device. The validated AF Effect on Quality of Life (AFEQT) was delivered at baseline (introduced 7 months after the study initiation).^18^ For the first 6 weeks, weekly surveys queried about procedural complications. Every week throughout the 1-year study period, participants were asked about recurrent AF events. Affirmative responses triggered more detailed questions, including the nature and care of any related complications and/or whether symptoms were present, and whether the AF was documented or diagnosed by a health care professional. Every 4 weeks, and surveys specific to emergency department (ED) visits and hospitalization admissions (with details regarding each given an affirmative response) were included. And at 6 and 12 months, the AFEQT was repeated.

### Validation of self-reported outcomes

#### AF events with AliveCor ECG

A subset of participants who owned a KardiaMobile device (AliveCor) integrated their account with the VIBRANT-AF mobile app. These devices are capable of patient-initiated ECG recordings. All KardiaMobile-based ECGs that were not flagged as normal sinus rhythm were manually reviewed for the presence of AF and over-read by a board-certified cardiac electrophysiologist.

#### Complications and adverse events

We validated self-reported complications by conducting electronic health record review of all consented participants providing Health Insurance Portability and Accountability Act authorization at the UCSF (Supplemental Methods). Overall agreement, sensitivity, and specificity were calculated using a patient-level composite indicator and pooled complications.

### Statistical analysis

Normally distributed continuous variables are presented as mean (SD) and were compared using t-tests. Categorical variables were compared using the Fisher exact or chi-square test as appropriate. Proportions of patients reporting AF, ED visits, and hospitalization were calculated and plotted from those who replied to the surveys on the corresponding specific week/month.

The analysis was conducted after a minimum of 200 participants had completed 1-year of follow-up. Assuming a baseline prevalence of a given characteristic of 50%, we estimated that 200 participants would provide 80% power to detect a 20% difference in characteristics between approximately equal numbers of compliant and noncompliant participants using a 2-tailed alpha of 0.05.

To identify independent predictors of survey adherence defined as ≥ the median percent of surveys completed (50%) or response to at least 1 last month survey, 2 multivariable logistic regression models were conducted: first, in a hypothesis-free assessment, all baseline characteristics that were associated with adherence in unadjusted analyses with *P* values <0.10 were included; second, we tested the a priori hypothesis that evidence of initial engagement in at least 1 of the first 2 weeks surveys (demonstrating a participant’s ability to successfully download the app, receive texts, and at least initial interest in the study) would predict adherence throughout the study. Multivariable Cox proportional hazard regression models were used to examine predictors of time to first AF recurrence after employing a 3-month blanking period. Given that no unadjusted associations met the *P* < 0.10 threshold, variables with *P* values < 0.20 were included in multivariable models, and only covariates with the lowest *P* values were retained to assure that no more than 10 covariates per outcome were included.

Concordance between self-reported AF recurrence and device-detected AF during the 3-month blanking period and for 1-year follow-up was assessed using overall agreement and Cohen’s κ statistic. Directional disagreement was evaluated using the McNemar exact test. For validation of complications compared to medical documentation, overall agreement, sensitivity, and specificity were calculated using a patient-level composite indicator and pooled complications.

A complete-case analysis was performed due to the low frequency of baseline missing data. Statistical analyses were performed using R (version 4.2.1; R foundation for statistical computing). A 2-tailed *P* value <0.05 was considered statistically significant.

## Results

**Figure 1:**
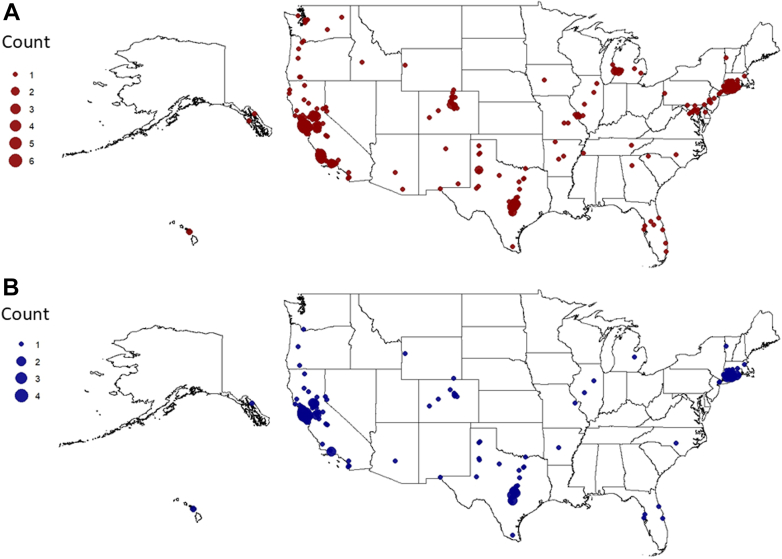
**Geographical Distribution of Locations of Participants Enrolled** (A) Participants that reached 6 weeks follow-up. (B) Participants that reached 1-year follow-up. Location based on zip code.

Between April 2022 and September 2024, 579 patients had downloaded the app and registered, and 539 (93%) from 17 sites ultimately consented for the study. At the time of the current analysis, 493 had completed 6-week follow-up and 236 patients completed 1-year follow-up (Supplemental Figure 1). Figure 1 illustrates the geographic distribution of participants over time, serving as a time-lapse snapshot that highlights the expanding reach of enrollment as new regions began contributing participants throughout the study period. As of the writing of this manuscript, 3 participants withdrew from the study (all after completing the 6 weeks follow-up).

Baseline characteristics of those that completed the study (1-year of follow-up) are presented in Table 1. Missing baseline data were infrequent (Supplemental Table 1). The median proportion of surveys completed throughout the study was 51% (IQR: 13% to 79%), and 50% completed at least 1 survey during the last (12th) month of study participation (Central Illustration), participants completed a median of 25 (IQR: 7-42) evisits (Supplemental Figure 4), and recurrent AF at 1 year was reported in 30% of participants. In a hypothesis-free multivariable analyses including only baseline variables, older age and the use of a consumer-based ECG device were independent predictors of a greater than median survey response rate throughout the study, whereas older age and nonsmoking status were independent predictors of retention and response at the last month of follow-up (Table 2).

**Table 1:** Baseline Characteristics by Adherence Level, Responded to More Than 50% of Surveys (>25 Weeks)

	Overall (N = 236)	Responded to More Than 50% of Surveys (n = 116)	Did Not Respond to More Than 50% of Surveys (n = 120)	*P* Value^a^
Age, mean (SD), y	56.5 (13.1)	58.0 (11.3)	55.0 (14.6)	0.08
Female, n (%)	72 (31)	39 (33)	33 (28)	0.31
Race, n (%)				0.33
White	212 (89.8)	108 (93.1)	104 (86.7)	
Black	1 (0.4)	0 (0)	0	
Asian/Pacific Islander	10 (4.2)	3 (2.6)	7 (5.8)	
Native American	2 (0.8)	0 (0)	2 (1.6)	
Other	11 (4.6)	5 (4.3)	6 (5)	
Hispanic, n (%)	21 (9)	5 (4.3)	16 (13.3)	0.11
Persistent AF, n (%)	65 (27.8)	35 (30.2)	30 (25.4)	0.42
Previous AF ablation, n (%)	67 (28.6)	31 (26.7)	35 (31.4)	0.48
ECG at home, n (%)	126 (53.3)	78 (67.2)	48 (40)	<0.001
Hypertension, n (%)	127 (54.3)	65 (56.0)	62 (52.5)	0.76
Diabetes, n (%)	22 (9.4)	14 (12.1)	8 (6.8)	0.24
CAD, n (%)	26 (11.1)	14 (12.1)	12 (10.2)	0.77
Previous MI, n (%)	13 (5.6)	7 (6.0)	6 (5.1)	0.79
CHF, n (%)	25 (10.7)	14 (12.1)	11 (9.3)	0.69
CHD, n (%)	12 (5.1)	4 (3.4)	4 (3.4)	0.51
Valve repair, n (%)	10 (4.3)	2 (1.7)	8 (6.8)	0.06
Implant, n (%)				0.78
None	207 (88.5)	102 (87.9)	105 (89)	
ILR	4 (1.7)	3 (2.6)	1 (0.8)	
Pacemaker	7 (3.0)	3 (2.6)	4 (3.4)	
ICD	12 (5.1)	6 (5.2)	6 (5.1)	
Other	3 (1.3)	1 (0.9)	2 (1.7)	
COPD, n (%)	12 (5.1)	5 (4.3)	7 (5.9)	0.85
Hypercholesteremia, n (%)	118 (50.4)	62 (53.4)	56 (47.5)	0.21
Previous stroke, n (%)	23 (9.8)	12 (10.3)	11 (9.3)	0.59
Sleep apnea, n (%)	92 (39.3)	51 (44.0)	41 (34.7)	0.35
Education, n (%)				0.22
No degree	35 (14.8)	17 (14.7)	18 (15)	
Undergraduate	99 (41.9)	56 (48.3)	43 (35.8)	
Master and above	75 (31.8)	33 (28.4)	42 (35)	
Other	25 (10.6)	10 (8.6)	15 (12.5)	
Unknown	2 (0.8)	0 (0.0)	2 (1.6)	
Unemployed, n (%)	8 (3.4)	2 (1.7)	6 (5.1)	0.31
Annual income, mean (SD), $1,000 USD	77 (22)	78 (19)	76 (25)	0.51
Current partner, n (%)				0.34
Never or other	17 (7.2)	9 (7.8)	8 (6.7)	
Currently with partner	178 (75.4)	91 (78.4)	87 (72.5)	
Separate/divorce/widow	39 (16.5)	16 (13.8)	23 (19.2)	
Unknown	2 (0.8)	0 (0.0)	2 (1.7)	
Smoked ever, n (%)	64 (31.7)	30 (26.3)	34 (38.6)	0.06
Consumed alcohol last year, n (%)	162 (76.4)	92 (80)	70 (72.2)	0.31
Completed at least one of the first 2 wks surveys, n (%)	182 (77.1)	103 (88.8)	79 (65.8)	<0.001

AF = atrial fibrillation; CAD = coronary artery disease; CHD denotes congenital heart disease; CHF = congestive heart failures; COPD = chronic obstructive pulmonary disease; ECG = electrocardiogram; HF = heart failure; ICD = implantable cardioverter defibrillator; ILR = implantable loop recorder; MI = myocardial infarction.

a *P* values for the difference compared to those who did not respond.

**Central Illustration:**
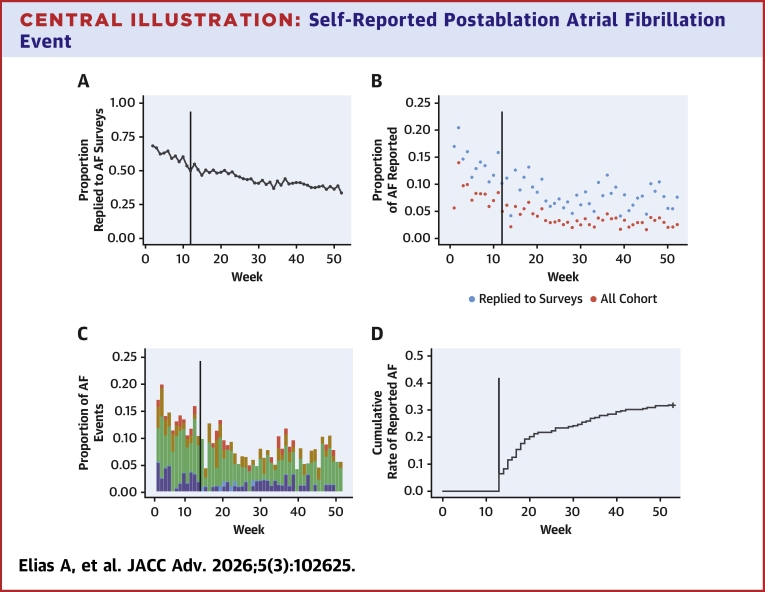
**Self-Reported Postablation Atrial Fibrillation Event** Among the 236 participants that had reached 1 year of follow-up as of the writing of the manuscript. (A) Response rates to the atrial fibrillation survey by week of follow-up. (B) Proportion of patients weekly self-reported atrial fibrillation from the complete cohort (red) and restricted to only those who replied to surveys (blue). (C) Proportion of self-reported atrial fibrillation by weeks and method of ascertainment, pink denotes regular ECG in a healthcare setting, green denotes ECG recording using a KardiaMobile, Apple Watch, or other handheld device, blue denotes a wearable ECG monitor prescribed by a healthcare provider, dark yellow denotes an irregular rate notification from a device such as an Apple Watch, purple denotes multiple methods of ascertainment. (D) Cumulative rates of self-reported atrial fibrillation (excluding events during the first 3 months). The black vertical line denotes the 3-month blanking period. AF = atrial fibrillation.

**Table 2:** Baseline Characteristics by Response to the Last Month Surveys

	Overall (N = 236)	Responded to the Last Month Surveys (n = 118)	Did Not Respond to the Last Month Surveys (n = 118)	*P* Value^a^
Age, mean (SD), y	56.5 (13.1)	58.2 (11.0)	54.8 (14.9)	0.044
Female, n (%)	72 (31)	38 (32)	34 (28.8)	0.57
Race, n (%)				0.39
White	212 (89.8)	108 (91.5)	104 (88.1)	
Black	1 (0.4)	0 (0)	1 (0.8)	
Asian/Pacific Islander	10 (4.2)	5 (4.2)	5 (4.2)	
Native American	2 (0.8)	0 (0)	2 (1.6)	
Other	11 (4.6)	5 (4.2)	6 (5)	
Hispanic, n (%)	21 (9)	7 (6)	14 (11.8)	0.26
Persistent AF, n (%)	65 (27.8)	38 (32.2)	27 (23.3)	0.13
Previous AF ablation, n (%)	67 (28.6)	29 (24.6)	38 (33.6)	0.22
ECG at home, n (%)	126 (53.3)	77 (65.2)	49 (41.5)	<0.001
Hypertension, n (%)	127 (54.3)	59 (50.0)	68 (58.6)	0.31
Diabetes, n (%)	22 (9.4)	13 (11.0)	9 (7.8)	0.42
CAD, n (%)	26 (11.1)	13 (11.0)	13 (11.2)	0.84
Previous MI, n (%)	13 (5.6)	6 (5.1)	7 (6.0)	0.22
CHF, n (%)	25 (10.7)	12 (10.2)	13 (11.2)	0.80
CHD, n (%)	12 (5.1)	6 (5.1)	6 (5.2)	1.00
Valve repair, n (%)	10 (4.3)	3 (2.5)	7 (6.0)	0.19
Implant, n (%)				0.27
None	207 (88.5)	106 (89.8)	101 (87.1)	
ILR	4 (1.7)	3 (2.5)	1 (0.8)	
Pacemaker	7 (3.0)	2 (1.7)	5 (4.3)	
ICD	12 (5.1)	6 (5.1)	6 (5.1)	
Other	3 (1.3)	0 (0.0)	3 (2.5)	
COPD, n (%)	12 (5.1)	7 (5.9)	5 (4.3)	0.31
Hypercholesteremia, n (%)	118 (50.4)	62 (52.5)	56 (48.3)	0.81
Previous stroke, n (%)	23 (9.8)	11 (9.3)	12 (10.3)	0.58
Sleep apnea, n (%)	92 (39.3)	51 (43.2)	41 (35.3)	0.46
Education, n (%)				0.66
No degree	35 (14.8)	16 (13.6)	19 (16.1)	
Undergraduate	99 (41.9)	51 (43.2)	48 (40.7)	
Master and above	75 (31.8)	38 (32.2)	37 (31.4)	
Other	25 (10.6)	13 (11.0)	12 (10.1)	
Unknown	2 (0.8)	0 (0.0)	2 (1.6)	
Unemployed, n (%)	8 (3.4)	2 (1.7)	6 (5.2)	0.28
Annual income, mean (SD), $1,000 USD	77 (22)	78 (20)	76 (24)	0.65
Current partner, n (%)				0.30
Never or other	17 (7.2)	9 (7.6)	8 (6.8)	
Currently with partner	178 (75.4)	93 (78.8)	85 (72.0)	
Separate/divorce/widow	39 (16.5)	16 (13.6)	23 (19.5)	
Unknown	2 (0.8)	0 (0.0)	2 (1.7)	
Smoked ever, n (%)	64 (31.7)	28 (24.3)	36 (41.4)	0.015
Consumed alcohol last year, n (%)	162 (76.4)	94 (80.3)	68 (71.6)	0.26
Completed at least one of the first 2 wks surveys, n (%)	182 (77.1)	105 (88.9)	77 (65.2)	<0.001

Abbreviations as in Table 1.

a *P* values for the difference compared to those who did not respond.

**Figure 2:**
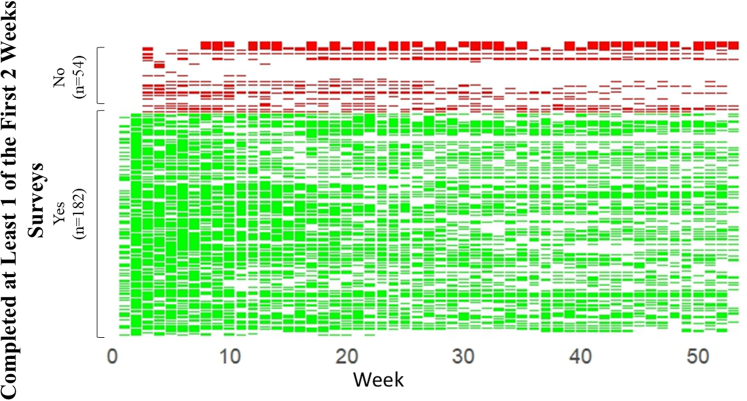
**Weekly Response Trends Stratified by Completion Status of First 2 Weeks Atrial Fibrillation Surveys** Weekly atrial fibrillation survey patterns among the 236 study participants who reached the 1-year follow-up are shown in the plot. Participants are grouped based on whether they completed either of the first 2 weeks' surveys: those who completed are marked in green, and those who did not are marked in red. Each horizontal line on the Y-axis represents a participant. Weeks with survey responses are filled with the corresponding color (green or red), whereas weeks without responses are left blank.

Among those that completed surveys in the first 2 weeks, more than 56% completed more than 50% of all surveys throughout the 1-year follow-up, and nearly 57% completed at least 1 survey during the last month of their study participation (Tables 1 and 2, Supplemental Table 2). Participants who completed at least 1 of the first 2 weeks surveys demonstrated greater adherence throughout the 1-year follow-up (Figure 2). In the prespecified multivariable model, adherence to at least 1 week survey during the first 2 weeks independently predicted a 2.5-fold increase in the odds of response by the last month and a 2-fold increase in the odds of responding to more than 50% of the surveys, although the latter did not achieve statistical significance (Table 3).

**Table 3:** Predictors of Surveys Adherence

	Responded >50% of AF Surveys (Hypothesis-Free Model)		Responded >50% of AF Surveys (Model Including Response to at Least 1 of the First 2 Wks AF Surveys)		Responded to Last Month AF Surveys (Hypothesis-Free Model)		Responded to Last Month AF Surveys (Model Including Response to at Least 1 of the First 2 wks AF Surveys)	
	OR (95% CI)	*P* Value	OR (95% CI)	*P* Value	OR (95% CI)	*P* Value	OR (95% CI)	*P* Value
Age (per 10 ys)	1.33 (1.05-1.71)	0.02	1.36 (1.07-1.75)	0.014	1.41 (1.11-1.82)	0.006	1.45 (1.13-1.88)	0.003
ECG at home	1.98 (1.09-3.6)	0.02	1.76 (0.95-3.25)	0.06	1.57 (0.87-2.86)	0.13	1.36 (0.73-2.52)	0.32
Valve repair	0.36 (0.05-1.79)	0.24	0.39 (0.05-1.95)	0.28	-	-	-	-
Smoked ever	0.58 (0.31-1.09)	0.09	0.58 (0.31-1.09)	0.09	0.42 (0.23-0.79)	0.008	0.42 (0.22-0.79)	0.008
Responded to at least to 1 of the first 2 wks AF surveys	-	-	2.18 (0.93-5.26)	0.07	-	-	2.53 (1.08-6.18)	0.03

Abbreviations as in Table 1.

**Figure 3:**
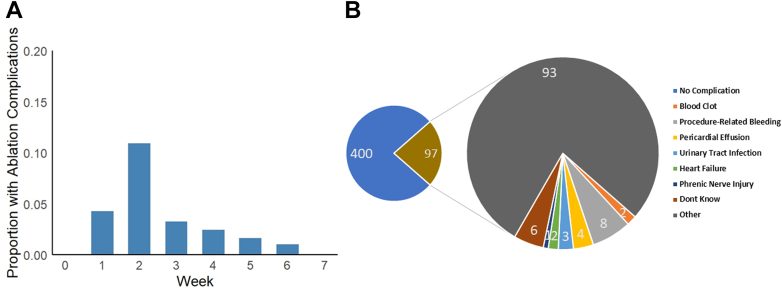
**Self-Reported Complications** Among the 493 participants that had reached at least 6 weeks of follow-up as of the writing of this manuscript. (A) Proportion of participants reporting at least one ablation-related complications by week. (B) Self-reported complication frequency by complication type.

Among the 493 participants who completed the 6-week postablation follow-up surveys, 97 (20%) reported at least 1 ablation-related complication for a total of 119 different complications (Figure 3). Of these, only 20 (4%) could be directly attributed to the catheter ablation procedure, including complications such as procedure-related bleeding, pericardial effusion, phrenic nerve injury, blood clots, urinary tract infections, and worsening of heart failure. The remaining 99 reported complications were nonspecific and were not clearly related to the catheter ablation procedure. In addition, a total of 405 adverse symptoms were reported by 203 participants without a concomitant diagnosis, including chest discomfort, swallowing difficulties, and fever (Supplemental Figure 2).

**Figure 4:**
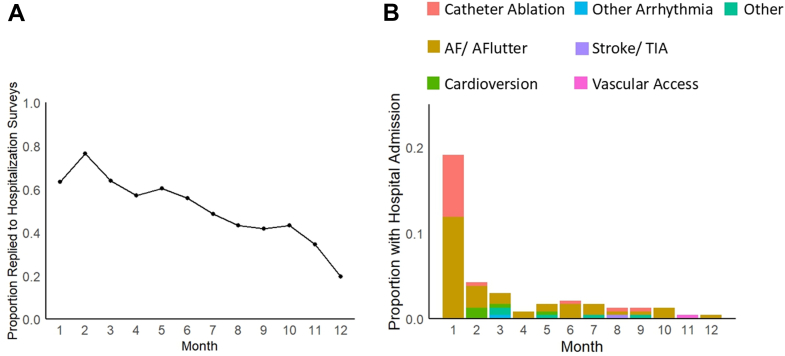
Self-Reported Hospitalizations Among the 236 participants that had reached 1 year of follow-up as of the writing of the manuscript. (A) Response to hospitalization surveys by month of follow-up. (B) Self-reported hospitalization proportion by week and admission reason. AF = atrial fibrillation; AFlutter = atrial flutter; TIA = transient ischemic attack.

The response rates to monthly ED and hospitalizations surveys were approximately 50% throughout the study as shown in Supplemental Figures 3 and 4, respectively. Among 236 of those who reached 1-year follow-up, 12 (5%) participants reported ED visits during follow-up, mostly related to episodes of AF or atrial flutter. The proportion of patients reporting a hospitalization and the related reasons are shown in Figure 4. AF and atrial flutter were the predominant causes of hospitalization, and the considerable proportion of hospitalizations during the first month could be explained by reporting the original index catheter ablation hospitalizations.

**Figure 5:**
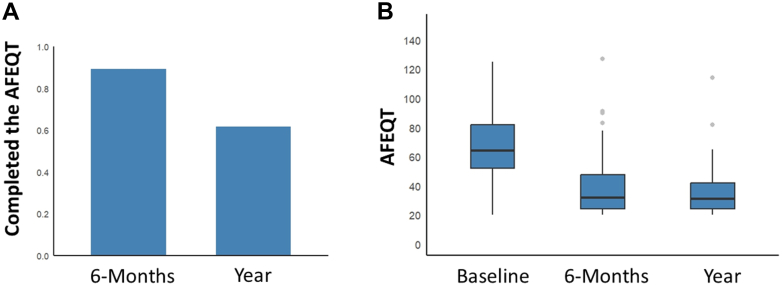
**Quality of Life Questionnaire by Atrial Fibrillation Effect on Quality of Life** Among the 141 participants offered and completed AFEQT questionnaire at baselines and that had reached 1 year of follow-up as of the writing of the manuscript. (A) Response to AFEQT questionnaires. (B) Boxplots for AFEQT scores at follow-up. The middle line represents the median, the bottom and top of each box indicate the 25th percentile and 75th percentile, respectively, the Y error bars indicate the upper adjacent value (75th percentile plus 1·5 × IQR), and the lower adjacent value (25th percentile minus 1·5 × IQR) and the dots represent outliers. AFEQT = Atrial Fibrillation Effect on Quality of Life.

The total AFEQT score improved from baseline to 6 months, with sustained improvement at 12 months (Figure 5).

Sixty-one participants with at least 3-months of follow-up and 38 participants with all 12-months of follow-up had connected an AliveCor KardiaMobile device. Of those participants, 22 (57.9%) were documented by AliveCor tracing to have a recurrent AF within 1 year of ablation and 20 (52.6%) self-reported having recurrent AF (Table 4). There was 89.5% agreement between self-reported and device-documented AF, with substantial concordance between the 2 measures (κ = 0.79; 95% CI: 0.47-1.0). None of the baseline characteristics were significantly associated with self-reported AF recurrence (Table 5).

**Table 4:** Validation of Self-Reported Atrial Fibrillation Recurrence

	Blanking Period (n = 61)	Postblanking Period (n = 38)	1 Year (n = 38)
Self-reported atrial fibrillation	30/61 (49.2%)	12/38 (31.6)	20/38 (52.6)
AliveCor-reported atrial fibrillation	18/61 (29.5%)	19/38 (50.0%)	22/38 (57.9%)
Overall agreement	49 (80.3%)	29 (76.3%)	34 (89.5%)
Cohen’s κ	0.60	0.53	0.79

**Table 5:** Multivariable Predictors of Recurrent Atrial Fibrillation

	Unadjusted Model		Adjusted Multivariable Model^a^	
	HR (95% CI)	*P* Value	HR (95% CI)	*P* Value
Age (per 10 y)	1.10 (0.92-1.33)	0.27	1.015 (0.99-1.036)	0.15
Female	1.04 (0.64-1.71)	0.85	-	-
Race (non-White)	0.74 (0.32-1.71)	0.49	-	-
Ethnicity (Hispanic)	0.54 (0.26-1.14)	0.11	0.67 (0.33-1.33)	0.25
Persistent AF	1.19 (0.73-1.95)	0.48	-	-
Previous AF ablation	0.71 (0.42-1.21)	0.21	0.73 (0.41-1.30)	0.29
CAD	1.40 (0.73-2.65)	0.16	1.38 (0.67-2.84)	0.38
Previous MI	0.92 (0.33-2.52)	0.86	-	
CHF	1.58 (0.83-3.00)	0.16	1.81 (0.94-3.49)	0.07
CHD	1.03 (0.37-2.82)	0.95	-	-
Valve repair	0.86 (0.27-2.74)	0.81	-	-
Hypertension	1.14 (0.71-1.81)	0.58	-	-
Diabetes	1.35 (0.67-2.71)	0.39	-	-
Sleep apnea	1.34 (0.83-2.18)	0.56	-	-
Smoked ever	0.69 (0.41-1.17)	0.17	0.60 (0.34-1.05)	0.07
Alcohol consumption within the year prior to enrollment	1.47 (0.83-2.60)	0.18	0.96 (0.53-1.72)	0.89

Analyses are limited to those who reached a year of study participation as of the writing of this manuscript (n = 236, 75 with recurrent AF after applying a 3-mo blanking period).

Abbreviations as in Table 1.

a Age and previous AF ablation were forced into the model, N = 202 in the multivariable model.

Health Insurance Portability and Accountability Act authorization was obtained from 106 of the 193 participants enrolled at UCSF. The self-reported sensitivity for the composite indicator of any complication was 80% in participants who completed more than 1 of 6 weekly surveys (n = 88) (Table 6), rising to 83.0% among individuals who completed 100% of the first 3 weekly surveys (n = 56) and to 87.5% for those who completed more than two-thirds of the total 6 weekly surveys (n = 51). Excluding subjective symptoms not expected to result electronic health record documentation of a complication per se (chest discomfort and dysphagia), the specificity for the composite of any complication among individuals who completed more than two-thirds of weekly surveys was 87.0%. The pooled specificity of self-reported events across complication types was 96.6%, increasing to 99.0% when chest discomfort and dysphagia were excluded.

**Table 6:** Validation of Self-Reported Complications

	Self-Reported Complications	Chart-Reported Complications	Overall Agreement
Any complication	49 (43.4)	26 (24.5)	66 (62.3)
Any complication (excluding chest discomfort or dysphagia)	17 (16.0)	15 (14.2)	88 (83.0)
Chest discomfort	38 (35.8)	12 (11.3)	75 (70.8)
Dysphagia	14 (13.2)	3 (2.8)	95 (89.6)
Pericardial effusion	1 (0.9)	1 (0.9)	106 (100)
Procedure-related bleeding	2 (1.9)	1 (0.9)	105 (99.1)
Heart failure	1 (0.9)	4 (3.8)	101 (95.3)
Fever	1 (0.9)	3 (2.8)	104 (98.1)
Urinary tract infection	1 (0.9)	2 (1.9)	105 (99.1)
Phrenic nerve injury	1 (0.9)	0 (0)	105 (99.1)
Other	12 (11.3)	7 (6.6)	91 (85.8)

Values are n (%). 106 records reviewed.

## Discussion

A patient-facing mobile app appeared to provide a feasible tool to perform surveillance for effectiveness and complications after catheter ablation procedures for AF. The overall adherence rates were around 50%, although evidence of initial engagement was associated with a 2-fold increase in retention throughout 1-year follow-up. Other independent predictors of study adherence included older age and owning a consumer-based ECG recording device. Despite reliance on technology and smartphone ownership, there was no evidence that adherence rates were associated with patient income, race, or ethnicity. Despite only 50% weekly compliance, most participants engaged in more than 25 separate “evisits” (or assessments) over time.

QA/QI measures are essential for maintaining efficient health care systems in general,^19^ as well in electrophysiology.^20^^,^^21^ Adopting such a scalable platform could provide an opportunity to collect extensive data on the effectiveness and safety of such procedures in a cost and resource-efficient fashion. In fact, enrolling participants from states outside the collaborating centers, or even from states that were not adjacent to collaborating centers such as Hawaii, Florida, or Alaska, highlights the potential of this promising platform.

Although this current assessment of feasibility was not designed to validate self-reported rates of AF recurrence or complications, the similarity of the observed proportions compared to previously published data using conventional means of outcome ascertainment suggests the reports may be accurate.^22^, ^23^, ^24^, ^25^ In fact, we have previously validated self-report of AF as highly accurate using remote-based studies,^26^ and other investigations, including studies using the Eureka Digital Research Platform, have similarly found self-report of arrhythmias, health care utilization, and complications to be reasonably valid.^17^^,^^27^^,^^28^ A subset of current participants provided evidence of reasonable agreement between self-reported AF and ECG strips obtained from remotely-connected KardiaMobile devices. It is worth noting that AF recurrence rates using this fully digital platform were similar to those observed in registries using more conventional means.^22^, ^23^, ^24^

The platform successfully achieved highly dense follow-up, with a median of 25 evisits at 1 year. To our knowledge, this represents the highest density of follow-up visits reported in AF catheter ablation registries,^22^, ^23^, ^24^ and even more frequent than clinical trials which typically have up to 6 office and telehealth visits combined.^29^^,^^30^

Early compliance, specifically within the first 2 weeks, was the strongest predictor of study retention. This finding aligns with established knowledge regarding run-in periods often employed in clinical research.^31^ The relatively seamlessness of recruitment in VIBRANT-AF, not requiring personnel to explain the study in detail nor to obtain consent (all performed via the mobile app) may be a situation where such relationships are especially pronounced. This observation may be an important “lesson-learned,” particularly when considering applying remote and mobile app–based methods to clinical trials or other scenarios where retention is especially pertinent.

Contrary to the common perception that only younger individuals may be “tech-savvy” enough to use a mobile app, our multivariable analyses demonstrated that older age was an independent predictor of ongoing engagement, perhaps reflecting greater interest or availability to participate in the study. In addition, participants who owned a consumer ECG device were more likely to complete their surveys, suggesting that other evidence of being more health conscious and potentially more interested in consumer health technologies might help identify ideal candidates for studies such as VIBRANT-AF.

Unlike the great majority of conventional research, participants were consented and subsequently remained engaged solely through the mobile platform, which could provide substantial cost-savings for prospective research data collection. Notably, as has been demonstrated in other “hybrid” trials,^32^, ^33^, ^34^ research personal can monitor participant compliance and retention automatically, and expend effort only among those who fail to respond to surveys. In addition to the importance of having a digitally scalable tool to assess the effectiveness of AF catheter ablation after the blanking period, weekly self-reported AF surveys might serve as a clinical logbook for AF. This approach could be a more reliable than depending solely on snapshot ECG logs or patients’ memories of symptoms or events later when they are seen in clinic. Such surveys could provide a more comprehensive, real-time summary of symptomatic AF episodes, helping clinicians make informed decisions about the need for and the timing of antiarrhythmic drugs or repeat ablation.

Self-reported procedural complications, such as bleeding, pericardial effusion, and embolic events, were consistent with those previously reported in studies using conventional means of outcome ascertainment.^25^^,^^35^ Although such reports are likely to be imperfect, they provide a proof-of-concept regarding a feasibly (and potentially inexpensively) tool to monitor for complications once a patient has left a referral center. Furthermore, the registry captured symptoms such as chest discomfort, swallowing difficulties, and fever. This information could be valuable in understanding the early diagnostic significance of these symptoms for rare but serious or fatal complications, such as atrioesophageal fistula and pulmonary vein stenosis.^36^^,^^37^

A validation of self-reported and electronic health record–determined complications demonstrated good overall agreement, sensitivity, and specificity. The sensitivity for patient reports of chart-documented complications improved with increasing rates of survey completion. Participants were more likely to report subjective chest discomfort or difficulty swallowing than was documented on electronic medical record review—this is to be expected given that such symptoms may not rise to the level of a health care visit or medical documentation, in fact demonstrating the heightened sensitivity of patient-facing reports of such postablation symptoms. Conversely, electronic medical record review was more likely to identify heart failure exacerbations requiring escalation of diuretic agents, fever, and urinary tract infection, demonstrating limitations perhaps inherent to patient knowledge or communication of the nature of such events from health care professionals back to their patients. Although routine follow-up of quality of life measures is important, it is generally not feasible outside the context of clinical trials and prospective registries.^38^ Specifically, collecting data such as an AFEQT score generally requires personnel time, meaning the time of a paid nurse or other hospital personnel.

Although mobile apps have been used to help monitor recurrent AF in various settings^39^^,^^40^ (including after ablation),^41^ to our knowledge no mobile app has been produced that provides an all-inclusive tool for patient informed consent, collection of data related to lifestyle, complications, and recurrent AF, and in a fashion that allows for seamless enrollment across multiple centers with minimal effort from research personnel. Indeed, in the post-AF ablation context, it is especially crucial to monitor for potential complications to facilitate quality assurance and improvement as recommended in professional societal statements.^14^^,^^15^ In fact, a scientific statement from the American Heart Association included implementing effective, widely accepted, cost-effective, and time-efficient mHealth interventions to improve cardiovascular health as a top health priority.^42^

Telehealth represents a complementary approach for postablation monitoring, allowing ongoing follow-up with the performing center for symptoms and quality of life. Although it was not employed or evaluated in the current study, future research should explore integrating mobile applications with telehealth to reinforce conventional patient monitoring and support broader scalability.

### Study limitations

It is important to acknowledge several limitations. The adherence rates for the surveys were moderate, and methods to improve adherence are needed to harness maximal capabilities offered by such platform. However, this is not a limitation of this observational study per se (which can only report what actually occurred), but rather of the platform as currently constructed. Moreover, the median number of evisits exceeded the number of visits in clinical practice, even the number of visits in major clinical trials and prospective registries.^22^, ^23^, ^24^^,^^29^^,^^30^ The fact that adherence was substantially higher among those who were initially engaged may suggest that a run-in period,^31^ particularly for interventional studies or randomized trials that leverage a mobile app–based approach, may be prudent. It is also worth mentioning that such a digital platform may complement conventional means, such as spending resources using conventional research personnel only for those who are not compliant (which may then still reduce the cost of conducting a study in this population by 50%). As mentioned, although validation of event accuracy was performed for a subset of patients in this report, validating these self-reported events in a larger cohort will be an important next step. However, some patient-reported outcomes, such as the AFEQT, can by definition only be determined by self-report, potentially highlighting a particularly valuable niche for this sort of approach. The current method is limited to those who own a smartphone and are able to use mobile apps. Of interest, 90% of the general population is now thought to own a smartphone,^43^ and, contrary to the notion that only young tech-savvy individuals may be able to participate in such a study, we actually found that older age predicted better retention. Because enrollment was designed to rely heavily on the mobile app itself and not on research personnel, we were unable to comment on the number or characteristics of individuals screened to identify predictors of those more or less likely to participate beyond the proportion of those who downloaded the app that ultimately consented. We acknowledge there was likely selection bias given the requirement to own a smartphone and in light of the relatively high socioeconomic status of participants. Engagement declined over time, and participants who dropped out may have had lower quality of life, introducing potential differential misclassification that could bias follow-up estimates toward higher measured AFEQT scores. Finally, patients who did not speak either English or Spanish were not included, and therefore it may be inappropriate to extend our findings beyond those populations.

## Conclusions

Self-reported surveillance using a direct patient-facing mobile app after catheter ablation is feasible. Such a platform could provide a cost-efficient and scalable tool for QA and QI efforts and may facilitate the conduct of observational studies and randomized trials, although its scalability and generalizability may be limited by smartphone ownership, mobile app literacy, and the selective characteristics of the enrolled population.

Perspectives **COMPETENCY IN MEDICAL KNOWLEDGE:** Mobile application–based patient surveillance is feasible following catheter ablation for AF, even for participants located far from collaborating centers.

**COMPETENCY IN PATIENT CARE:** Self-reported rates of AF recurrence and complications were consistent with previous reports using conventional means.

**TRANSLATIONAL OUTLOOK:** Such a platform could provide a cost-efficient and scalable tool for QA and QI efforts and may facilitate and provide an adjunct tool to conventional methods used to conduct observational studies and randomized trials, particularly for patient-reported outcomes.

## Funding support and author disclosures

This trial was funded by 10.13039/100000002National Institute of Health (NIH) grant R01 HL158825. Dr Hsu has received honoraria from 10.13039/100016304Medtronic, 10.13039/100000046Abbott, 10.13039/100008497Boston Scientific, 10.13039/501100005035Biotronik, 10.13039/100008897Janssen Pharmaceuticals, 10.13039/100002491Bristol-Myers Squibb, 10.13039/100004319Pfizer, 10.13039/100004339Sanofi, Altathera Pharmaceuticals, Milestone Pharmaceuticals, 10.13039/100015345Zoll Medical, 10.13039/100006983iRhythm, 10.13039/100006520Edwards Lifesciences, Viz.ai, and Biosense-Webster; and has equity interest in Vektor Medical. Dr Freeman has received research funding from the National Institutes of Health and the American College of Cardiology National Cardiovascular Data Registry; and has served as a consultant/advisor for Boston Scientific, Abbott, Johnson & Johnson, PaceMate, and Medtronic. All other authors have reported that they have no relationships relevant to the contents of this paper to disclose.
