# Supplementary material for: Feasibility of Self-Reported Surveillance After Catheter Ablation for Atrial Fibrillation Using A Mobile Application
Source: JACC Adv. 2026 Mar 25;5(3):102625. doi: 10.1016/j.jacadv.2026.102625 (PMC13352016; doi:10.1016/j.jacadv.2026.102625)
Supplement: Supplementary Material [file mmc3.docx]

**Supplemental Material**

**Supplemental Figure 1. STROBE Flow Diagram of Analysis of the VIBRANT-AF Analysis.**


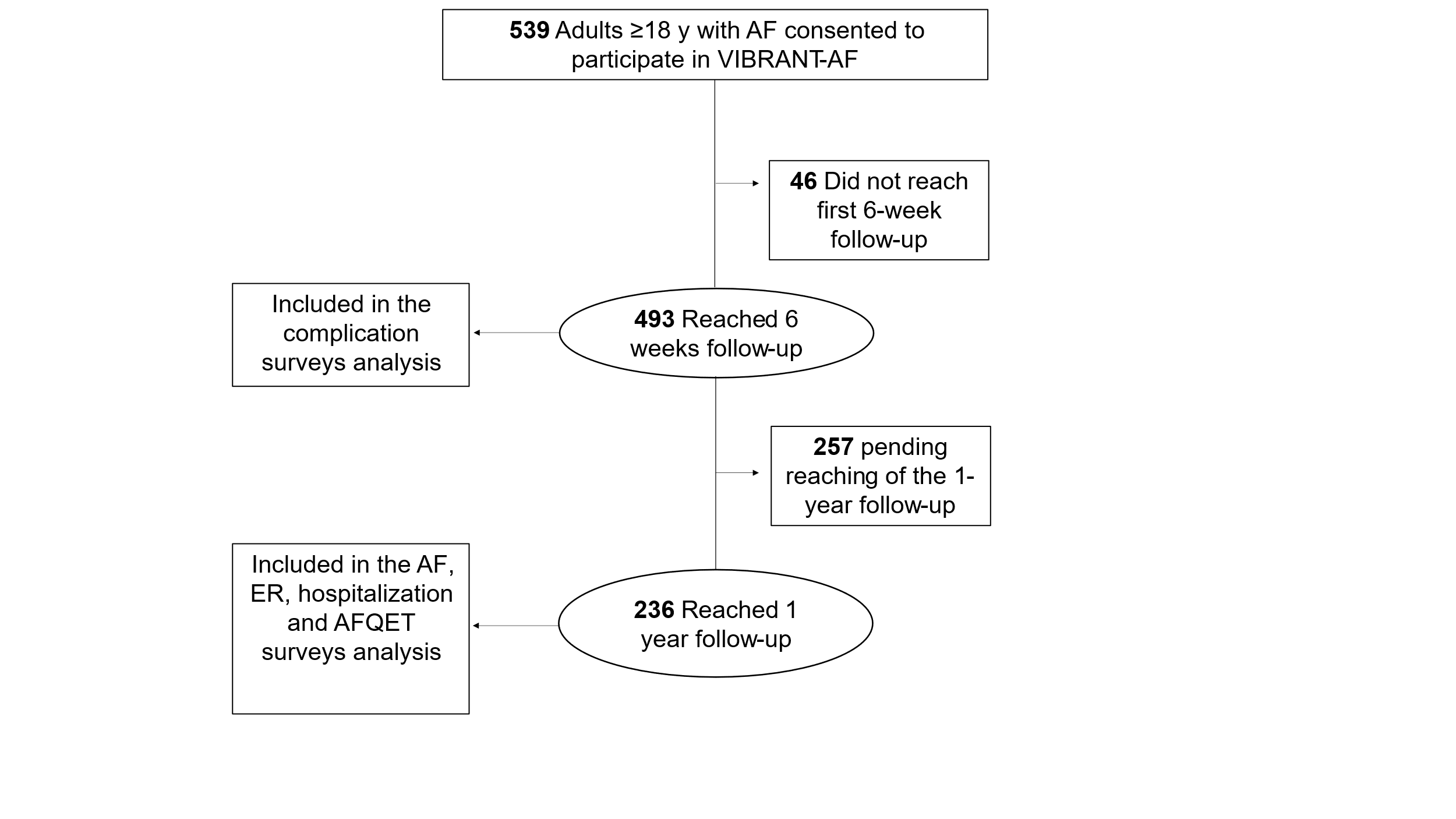


**Supplemental Figure 2. Self-Reported Symptoms and Signs**


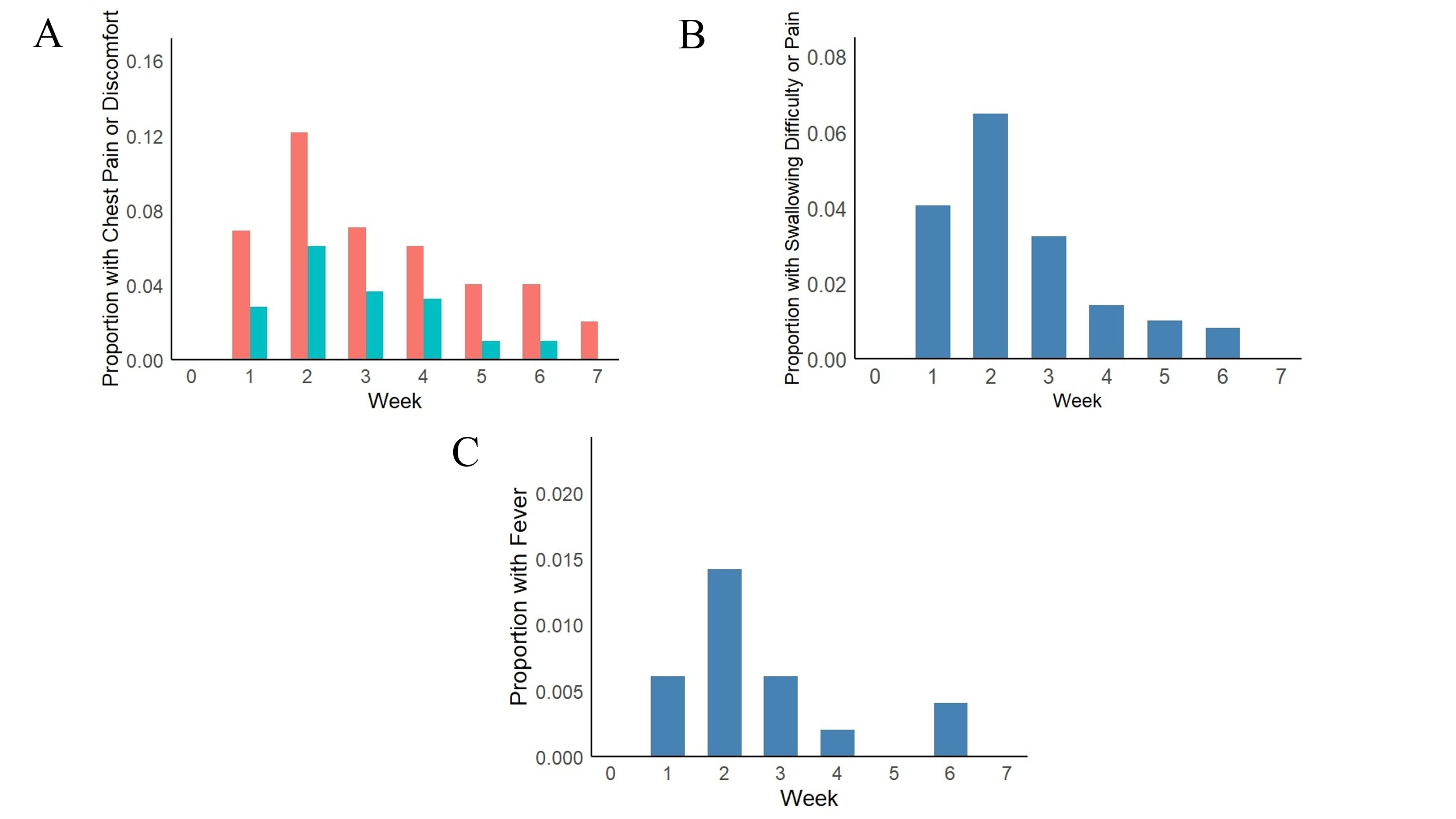


Among the 493 participants that had reached at least 6 weeks of follow-up as of the writing of this manuscript.

**Panel A**: Proportion reporting chest pain or discomfort by week, turquoise denotes pleuritic, pink denotes non-pleuritic.

**Panel B**: Proportion reporting difficulty or painful swallowing by week.

**Panel C**: Proportion reporting fever, defined as temperature ≥ 101.3 degrees Fahrenheit or ≥ 38.5 degree Celsius.

**Supplemental Figure 3. Self-Reported Emergency Department Visits**


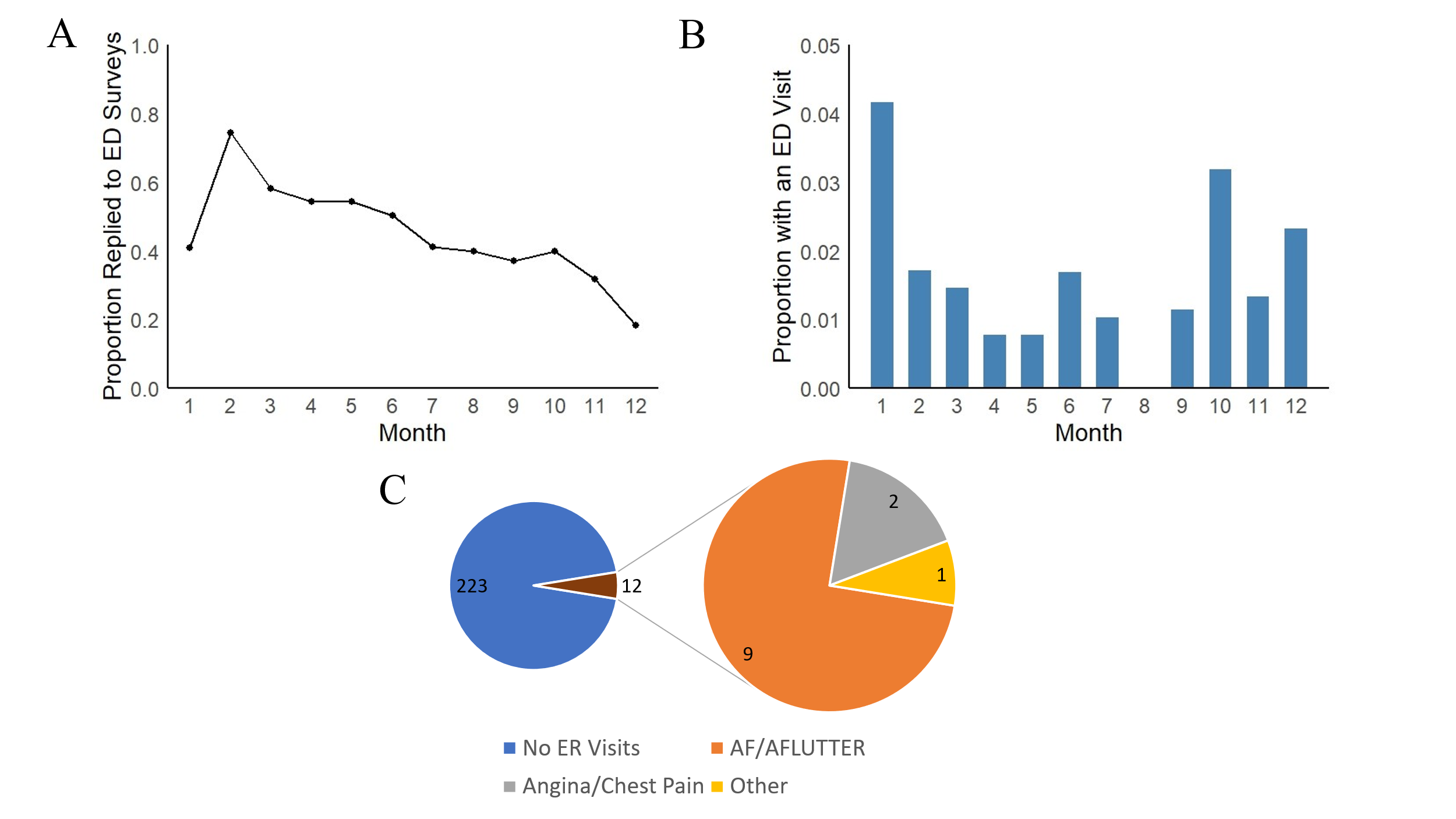
Among the 236 participants that had reached one year of follow-up as of the writing of the manuscript.

**Panel A**: Proportion of participants that responded to the monthly ED surveys.

**Panel B**: Proportion of participants reporting an ED visit by month.

**Panel C**: Number of self-reported ED visits by admission reason over the study period.

**Supplemental Figure 4. Distribution of Survey Responses per Participant**
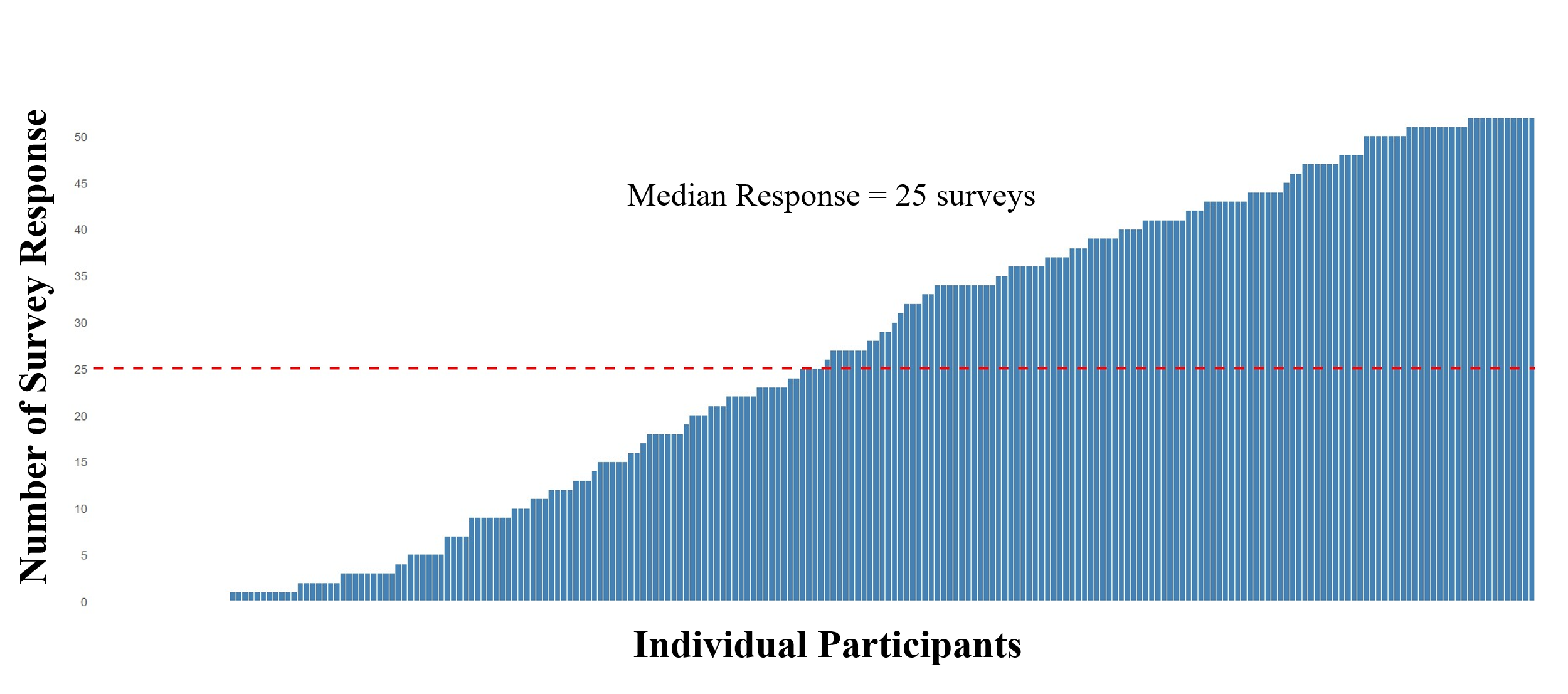


Distribution of the number of surveys replied per participant. Median = 25 surveys; first quartile (Q1) = 7; third quartile (Q3) = 42; mean = 25.

**Supplemental Table 1. Frequency of Missing Data by Variable**

|  | **Missing Data** |
| --- | --- |
|  | N=236 |
| **Age** – no. (%) | 0 |
| **Female** – no. (%) | 0 |
| **Race** – no. (%) |  |
| **Hispanic** – no. (%) | 0 |
| **Persistent AF** – no. (%) | 2 (0.8) |
| **Previous AF Ablation** – no. (%) | 2 (0.8) |
| **ECG at Home** – no. (%) | 0 |
| **Hypertension** – no. (%) | 2 (0.8) |
| **Diabetes** – no. (%) | 2 (0.8) |
| **CAD** – no. (%) | 2 (0.8) |
| **Previous MI** – no. (%) | 2 (0.8) |
| **CHF** – no. (%) | 2 (0.8) |
| **CHD** – no. (%) | 2 (0.8) |
| **Valve Repair** – no. (%) | 2 (0.8) |
| **Implant** – no. (%) | 2 (0.8) |
| **COPD** – no. (%) | 2 (0.8) |
| **Hypercholesteremia** – no. (%) | 2 (0.8) |
| **Previous Stroke** – no. (%) | 2 (0.8) |
| **Sleep Apnea** – no. (%) | 2 (0.8) |
| **Education** – no. (%) | 0 |
| **Unemployed** – no. (%) | 2 (0.8) |
| **Annual Income** – no. (%) | 0 |
| **Current Partner –** no. (%) | 0 |
| **Smoked Ever** – no. (%) | 34 (14.4) |
| **Consumed Alcohol Last Year** – no. (%) | 24 (10.2) |

**Supplemental Table 2. Concordance Between Patients Who Responded to More Than 50% of Surveys and the Last-Month Surveys**

|  | Responded to More Than 50% of Surveys | Did not Respond to More Than 50% of Surveys |
| --- | --- | --- |
| Responded to the Last Month Surveys | 97 (41%) | 21 (8.9%) |
| Responded to the Last Month Surveys | 18 (7.6%) | 99 (42%) |

Overall Concordance 83%

**Supplemental Methods:**

The medical record was reviewed for the presence of complications during the first six weeks following the procedural date, including events prior to and after discharge. Adverse events were categorized by the following definitions: (1) Procedural Bleeding: clinically significant bleeding requiring transfusion or subsequent clinic visit, imaging study, or procedure; (2) Stroke or TIA: clinical evidence of transient or permanent neurologic deficit with or without an imaging correlate, corroborated by a health professional; (3) Pericarditis: post-procedural chest discomfort requiring treatment with medications including colchicine, NSAIDs, or aspirin; (4) Pericardial Effusion: echocardiographic evidence of pericardial fluid that was not present pre-procedure; (5) Dysphagia: clinically significant difficulty or pain swallowing requiring communication with a medical staff member, medical treatment, or an imaging study; (6) Thromboembolism: blood clot identified by ultrasound or other imaging modality; (7) Fever: documented temperature greater than 101.3 degree F; (8) Urinary Tract Infection: dysuria or urinary frequency accompanied by a positive urinalysis requiring antibiotic therapy; (9) Pneumonia: shortness of breath or cough accompanied by radiographic findings consistent with pneumonia; (10) Phrenic Nerve Injury: clinical suspicion of phrenic nerve injury that manifests as dyspnea with or without hemidiaphragm elevation; (11) Left Atrial Esophageal Fistula; or (12) Pulmonary Vein Stenosis. Other complications included refractory headache, skin and soft tissue infection requiring treatment with antibiotics, and abscess requiring imaging and/or treatment with antibiotics. A participant-level composite indicator for any complication was created, defined as having experienced at least one complication during the first 6-weeks following ablation. Pooled sensitivity and specificity were also assessed as the proportion of chart-documented complications reported by patients, pooled across all complication subtypes. Subjective reports of chest discomfort and dysphagia were included and excluded as sensitivity analyses. Overall agreement was defined by concordant positive and concordant negative responses divided by the total number of participants reviewed. Sensitivity and specificity were assessed using completeness-restricted analyses to assess the impact of missing survey data on concordance.
